# Transforming meat waste into sustainable corneal keratoplasty models

**DOI:** 10.3389/fbioe.2025.1572127

**Published:** 2025-04-14

**Authors:** Ayman Mobin, Zayd Hashem, Peter R. Corridon

**Affiliations:** ^1^ Department of Biomedical Engineering and Biotechnology, College of Medicine and Health Sciences, Khalifa University of Science and Technology, Abu Dhabi, United Arab Emirates; ^2^ Department of Biomedical Engineering and Biotechnology, Khalifa University, Abu Dhabi, United Arab Emirates; ^3^ Healthcare Engineering Innovation Center, Khalifa University, Abu Dhabi, United Arab Emirates; ^4^ Center for Biotechnology, Khalifa University of Science and Technology, Abu Dhabi, United Arab Emirates

**Keywords:** sustainable tissue engineering, meat waste, decellularized ECM (dECM), urine-derived cells (UCs), recellularization, keratoplasty

## Abstract

With a rapidly global population, there is a critical need to enhance food production and waste management. This necessity is driving opportunities for sustainable integrated food chains committed to biovalorization and circular bioeconomic practices. One approach that aligns with this vision relies on sustainable tissue engineering, which offers opportunities to leverage food systems in the search for natural biomaterials from agricultural waste. In this perspective, we propose utilizing common meat waste sources, often associated with a high environmental footprint, to develop tissue graft models. These models reduce agricultural waste, decrease the reliance on animal testing, and support both biovalorization and medical innovation. Specifically, we explore a unique approach to generate corneal transplantation models completely from discarded components of the meat food chain, using the eyes and bladders. This strategy involves creating keratoplasty models by reseeding the decellularized extracellular matrix (dECM), encompassing three major corneal regions: the epithelium, stroma, and endothelium. Interestingly, these scaffolds can be recellularized with cellular lineages derived from stem niches harvested from urine. This approach integrates waste management with regenerative medicine, fostering sustainable advancements in tissue engineering.

## Introduction

Agri-food systems utilize various extraction, processing, storage, and distribution practices to support human consumption. Unfortunately, the recent pandemic, rapid population growth, climate change, ecological disasters, and political tensions emphasize a need for reassessments (McGreevy et al., 2022). These reassessments emphasize sustainability and waste management to help avert future disasters and conflicts.

These approaches aim to enhance food production through better-integrated food chains committed to biovalorization and circular bioeconomic practices. Several studies have outlined how sustainable integrated food chains can, in general, drive the conversion of organic waste and biomass into valuable products and consumption cycles (Ashokkumar et al., 2022) and specifically advance tissue engineering that supports cultured meat (Mancini et al., 2022; Manning et al., 2023; Perreault et al., 2023; Ramachandraiah, 2021; Hong et al., 2021) and biomaterials research (Khan et al., 2023; Tarafdar et al., 2021; Limeneh et al., 2022; Shibru et al., 2024).

Within the past decade, it was stated that roughly 10% of the 285 million vision-impaired people worldwide suffer from corneal opacities (He et al., 2020). Furthermore, roughly 2 million of the world’s 39.3 million population who are visually impaired can trace their cause of ailment to a corneal opacity (Wang E. Y. et al., 2023). The increasing prevenance of diseases, fungal, bacterial, viral, and metabolic conditions also contribute to chronic and end-stage conditions.

For instance, the rapid increase in the prevalence of diabetes mellitus has led to more cases of keratopathy and corneal neuropathy. Paradoxically, the SARS-CoV-2/COVID-19 vaccination presented adverse corneal events such as native corneal fiber neuropathy in COVID-19 patients (Yin et al., 2022). Such identified health issues underscore the necessity for further research to address corneal impairment.

As a means to simultaneously find novel solutions to these pressing issues, in this perspective, we propose utilizing common meat waste sources, often associated with a high environmental footprint, to develop tissue graft models. These models aim to reduce agricultural waste, decrease the reliance on animal testing, and support both biovalorization and medical innovation as we strive to address the global shortage of transplantable corneal tissues.

## The role of biomaterials in addressing the corneal transplantation shortage

**FIGURE 1:**
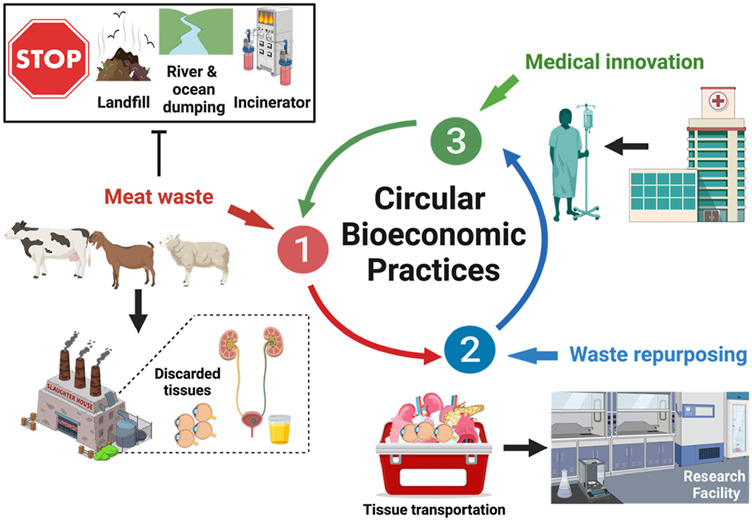
Repurposing Meat Waste to Drive Circular Bioeconomic Practices. Cyclic innovation can potentially arise from the collection of meat waste, including corneas and bladders, from local slaughterhouses. This process continues within the laboratory environment, where corneal tissue extraction and decellularization occur to generate viable scaffolds. These scaffolds can then be recellularized with cells generated from urine-derived cells. Ultimately, this process can be translated to clinical settings, where discarded human corneal tissues and patient-specific urine can be collected to generate viable keratoplasty grafts.

Biomaterials research involves the development of novel components and cutting-edge synthesis and fabrication technologies for industrial and clinical applications. A major clinical application is the development of transplantable organs and tissues, such as the cornea, to help the estimated 12.7 million worldwide who need keratoplasty (Liu et al., 2022). Recent evidence suggests the potential to improve xenografting technologies and models. Such models can be generated from repurposed human and animal ocular tissues, offering a novel pathway to utilize discarded by-products of the food industry for therapeutic purposes (Pantic et al., 2023; Wang et al., 2022; Wang et al., 2023a; Shibru et al., 2023), as illustrated in Figure 1.

## Advances in corneal substitutes

The cornea is a thin, transparent tissue for ophthalmic protection and light transmission. Prolonged exposure to natural elements, combined with the increasing prevalence of diseases, can cause irreversible complications, such as clouding, distortion, scarring, and eventual blindness. Current treatments for corneal repair include eye drops, ointments, oral medications, intraocular ring implantations, and, ultimately, transplantation. Although transplantation is the ideal correction for end-stage conditions, the limited availability of graft tissues necessitates new treatment avenues.

Significant advances in regenerative medicine have led to the development of corneal substitutes from bovine, caprine, ovine, porcine, and human cadaveric tissues through techniques like additive manufacturing (Jia et al., 2023), decellularized extracellular matrix (dECM) (Nara et al., 2016; Polisetti et al., 2021), and stem cell technologies (El Zarif et al., 2020). Recently, the potential of non-invasively sourced stem cells, including menstrual, adipose, and urine-derived cells (UCs), has been investigated for regenerative applications. UCs are highly proliferative and represent an unlimited and utterly non-invasive resource. These cells can be reprogrammed into iPSCs and differentiated into various cell lineages for corneal tissue engineering and regeneration (Jing et al., 2019).

## Repurposing slaughterhouse waste for sustainable keratoplasty models

**FIGURE 2:**
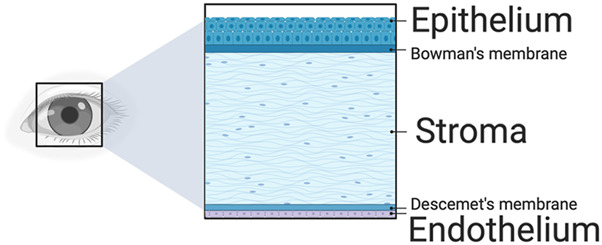
A schematic of the major corneal layers. The epithelium protects the cornea, enhancing its refractive power and triggering an immune response. Following this is the stroma, which offers crucial mechanical support and boasts the most potent refractive capability. The endothelium, the final layer, regulates water removal to maintain the cornea’s clarity. Additionally, the Bowman’s and Descemet’s membranes are between these three primary layers.

The cornea serves a dual role: it transmits light to the rod and cone cells and is a barrier against debris, bacteria, and other foreign bodies. Structurally, the cornea consists of three main layers-the epithelium, stroma, and endothelium - and two interfaces, namely, the Bowman’s layer and Descemet’s membrane, as shown in Figure 2. The epithelium, the outermost layer, through its inner basal cells, middle wing cells, and superficial squamous cells, enhances the eye’s barrier and refractive abilities (Yam et al., 2020). Additionally, this multilayered compartment accommodates bone marrow-derived ocular surface Langerhans antigen-presenting cells that regulate immunoreception and immunodefense.

Since the cornea is the eye’s outermost layer, it is especially prone to injuries. Trauma to the cornea can present as abrasions and keratitis, with more advanced damage causing corneal clouding, distortion, scarring, and eventual blindness. While it heals from minor injuries with the migration, proliferation, and differentiation of healthy peripheral epithelial cells, keratinocytes, and endothelial cells, its avascular nature limits innate wound healing due to the decreased access to nutrients and immune cells necessary for repair. Consequently, more severe injuries require external treatment like eye drops containing antibiotics, steroids, oral medications, phototherapeutic keratectomy, and keratoplasty.

## Corneal bioengineering: generating scaffolds from discarded eyes and reprogramming waste-derived stem cells

### The dECM for corneal scaffolding

**FIGURE 3:**
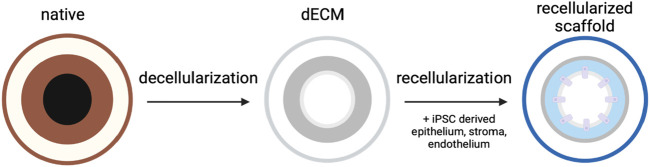
Utilizing the dECM for corneal scaffolding. An extracted cornea undergoes decellularization to create a dECM model. The dECM can then be repopulated with various patient-derived lineages to create a keratograft.

Advances in tissue engineering and regenerative medicine have led to studies investigating the use of the dECM as a tissue scaffold, as shown in Figure 3. Theoretically, these scaffolds maintain the ideal structural environment of the tissue and maintain the levels of endogenous cytokines and growth factors. The interaction between these elements of the ECM matrix with the surrounding cells plays a significant role in tissue differentiation, migration, adhesion, and proliferation (Chan and Leong, 2008; Chen et al., 2024). Furthermore, hydrogels that mimic ECM were shown to promote the proper proliferation and differentiation of ocular stem cells (Lu et al., 2024). Heparin sulfate is a vital component of the ocular ECM that promotes cell migration and proliferation. Other glycosaminoglycans, as well as different collagen types, have also been indicated to contribute to the ECM’s ability to promote cell differentiation (Yang et al., 2025). Analogs of heparin sulfate have shown promising clinical applications in restoring damaged ECM and, hence, restoring vision in patients suffering from corneal damage (Mahdavi et al., 2020). Providing an ECM-based scaffold with the required differentiation factors could play a vital role in advancing corneal repair and transplantation.

For instance, repurposing tissue waste from bovine animals has proven a worthy avenue to explore. In recent studies, corneas are collected from discarded sheep eyes (Pantic et al., 2023; Ali et al., 2024; Wang et al., 2023b), where they are subsequently decellularized to remove innate cellular components from the scaffolds. Then, the samples are immersed in glycerol to restore the opacity of the cornea. The scaffolds were tested and shown to have preserved ocular transparency, transmittance, and ECM microstructures necessary for proper corneal function. The result is a successfully decellularized corneal scaffold that maintains its microscopic integrity (Ahearne et al., 2020a). The unique value behind decellularized scaffolds is that they furnish an anatomical environment equipped with suitable cues for cell proliferation, differentiation, and organization (García-Gareta et al., 2020). Comparable studies involving human tissues have also provided evidence to support effective corneal decellularization/recellularization (Polisetti et al., 2021).

### Urine-derived cells (UCs) and their potential

One can argue that the ideal stem cells to repair corneal damage are the limbal epithelial stem cells and corneal stromal stem cells (Nurković et al., 2020). However, the limited availability, high costs, and challenges with cell isolation probe us to find other alternatives. In the past, biomaterials such as menstrual blood have been used to isolate stem cells; hence, we considered a more available and comparably radical biomaterial (Sanchez-Mata and Gonzalez-Muñoz, 2021). Urine, a non-invasive and potentially unlimited resource, contains several cells that can be reprogrammed to induce pluripotency and potentially support differentiation into cells of all three corneal germ layers (Jing et al., 2019).

Urine-derived cells primarily originate from the renal, bladder, and urethral epithelial linings and collectively comprise various cell types, including renal, blood, and immune cells. They also include an adult form of stem cells, whose differentiation potential is generally considered less than that of pluripotent stem cells (Sato et al., 2019).

### UC isolation, characterization and utilization

From a clinical perspective, such samples can be obtained from a prospective recipient to isolate the target cells. For this process, samples can be collected from routine urination or using a clean catch method to prevent the transmission of germs from the genitals (Diviney and Jaswon, 2021). For this latter process, it is advised to clean the genitals effectively, allow a small amount of initial urine to flow into the toilet, and then stop the flow and place the sterile collection tube to collect from the second stream of urine. The sterilized containers should contain antibiotics to inhibit bacterial growth (Burdeyron et al., 2020). The samples can be centrifuged at room temperature. Afterward, the supernatant can be aspirated and discarded to isolate the cell pellets. The pellets can be gently responded with sterile PBS and centrifuged again to remove loosely bound debris in the subsequent supernatant. The final pellet can then be cultured for downstream application, such as expansion, multi-/pluripotency verification, and differentiation with collagen- or gelatin-coated dishes/flasks, as extensively outlined in our previous work (Corridon et al., 2025).

**FIGURE 4:**
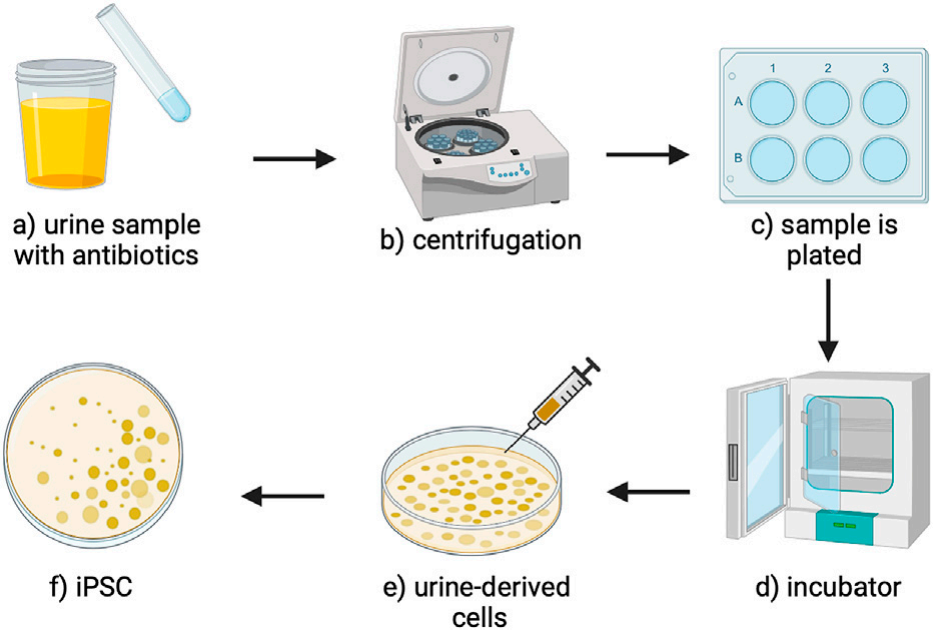
An overview of the UC extraction and reprogramming process. **(a)** The urine sample that can be aspirated from the bladder is mixed with antibiotics to sterilize the sample **(b)** then centrifuged to create a cell pellet. **(c)** The cell pellet is resuspended and plated. **(d)** The plates are incubated to culture urine-derived cells. **(e)** The urine-derived cells can be infected with retrovirus-producing Sox2, Klf4, Nanog, Oct4, and C-myc, reprograming the cells into **(f)** induced pluripotent stem cells (Schmidt and Plath, 2012; Aguirre et al., 2023).

**FIGURE 5:**
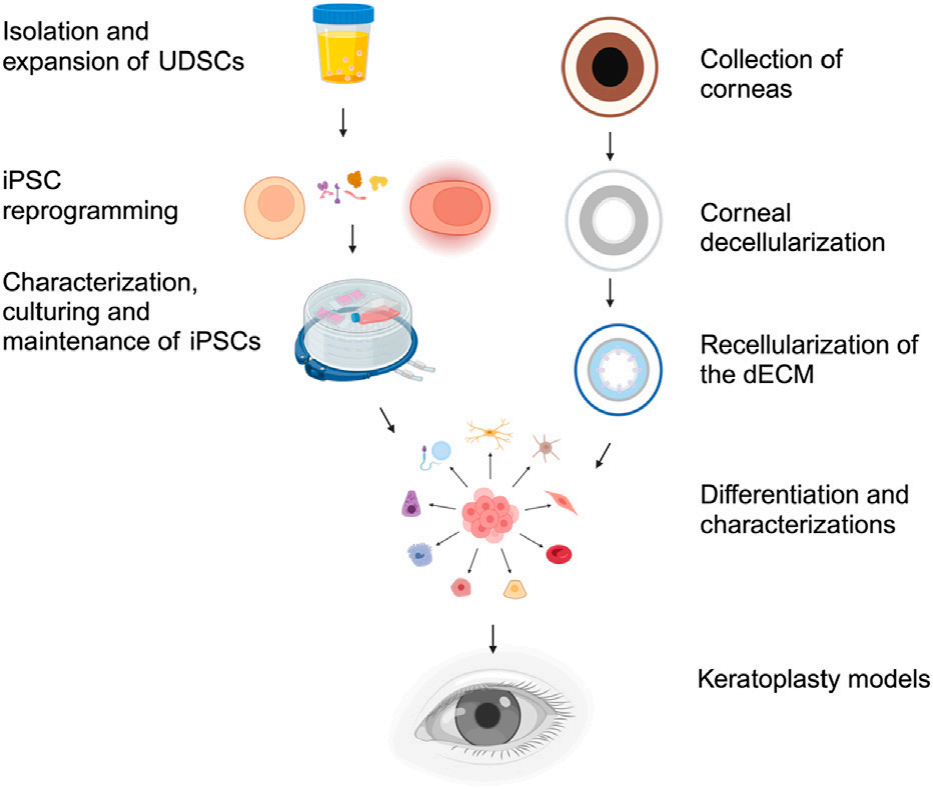
Generation of keratoplasty models from the dECM and UCs. After collecting and initial cellular reprogramming, iPSCs are characterized and cultured for further use. Various assays and tests are employed to characterize and confirm the pluripotency of the iPSCs. Simultaneously, corneas from either human or animal sources are collected and decellularized to create the dECM. The dECM is then tested for structural integrity and acellularity. The iPSCs are guided to differentiate into epithelial, corneal, and stromal stem cells by withdrawing pluripotency factors and adding signaling molecules and culture conditions that mimic the developmental cues for the desired cell lineages. Finally, the various compartments of the dECM are seeded with their appropriate differentiated lineages. Once seeded, the seeded dECM is ultimately tested to evaluate functional integrity via assays, including optical clarity, light transmissivity, refractive capacity, barrier function, and biocompatibility. The differentiated corneal cells can then be used for corneal tissue engineering and keratografting.

Notably, within 7–8 days, urine-derived cell colonies can be formed in the plate (Lang et al., 2013). Once the UCs are reprogrammed into IPSCs, presented in Figure 4, they must be assessed for their pluripotency, which can be done using immunostaining techniques for various biomarkers, including crucial transcription factors like Sox2, Klf4, Nanog, Oct4, and C-myc, as well as surface antigens such as SSEA3/-4, Tra-1-60, and Tra-1-81 (Baghbaderani et al., 2016), as outlined in Figure 5. After isolation and characterization, established differentiation protocols can be applied to derive epithelial, stromal, and endothelial lineages. These methods aligned with existing stem cell and corneal regeneration literature protocols (Corridon et al., 2025). Beyond this, colony expansion follows.

### Corneal decellularization and recellularization

Several approaches, as summarized in Table 1, have been defined throughout the literature to generate dECM scaffolds (Wang et al., 2023b; Procházková et al., 2024; Ahearne et al., 2020b; Fernández-Pérez and Ahearne, 2020; Isidan et al., 2019; Murtaza et al., 2024). These methods generally combine chemical and mechanical treatments, whereby extracted corneas can be agitated in a given agent, and swelling, a natural consequence of the decellularization process, can be limited using osmotic agents (like dextrans and glycerol) to preserve ultrastructural tissue properties. After which, structural and functional assays, like histology and spectroscopy, can be respectfully employed to gauge corneal structure and optical function.

**TABLE 1:** Summary of the corneal decellularization approaches. This table outlines commonly used classes of decellularization agents in corneal tissue processing, providing representative examples, their functional roles, and practical considerations, particularly regarding extracellular matrix (ECM) preservation and optical transparency.

Class of agent	Type (examples)	Role in corneal decellularization	Outcome
Detergents	Triton X-100, SDS, SDC, Tween-20/80, CHAPS	Disrupt lipid membranes and lyse cells	Non-ionic detergents (e.g., Triton X-100) preserve ECM; ionic detergents (e.g., SDS) are harsher but more effective for nuclear removal; and non-ionic zwitterionic detergents preserve ECM and transparency
Enzymes and Chelators	DNase I, RNase A, Trypsin, EDTA, EGTA	Degrade nucleic acids and disrupt cell–matrix or cell–cell adhesion	Often used after detergents to clean residual cell components; chelators enhance enzymatic access
Osmotic Agents	NaCl (hypertonic/hypotonic), deionized water	Induce osmotic shock to rupture cells	Used as pre-treatment, rinse, or in cycles with detergents
Chemical Disruptors (Acids/Bases/Oxidizers)	Peracetic acid, HCl, acetic acid, NH_4_OH, NaOH, H_2_O_2_	Additional lysis, sterilization, or nuclear denaturation	Strong agents; use at low concentrations to avoid ECM and transparency loss

In comparison, for scaffold recellularization, various additional aspects must be considered, including cell seeding density, incubation periods, and compartment-specific reseeding of epithelial, stromal, and endothelial layers. Various approaches have been evaluated, and novel ones are being considered; however, as we have previously postulated, we believe a combination of approaches will be needed to support the regeneration of dedicated epithelial and endothelial layers while simultaneously facilitating sparse yet widespread cell populations in the stroma. Specifically, various cell plating- and injection-based reseeding techniques can be applied to reintroduce the various cell lineages into their respective compartments. Chemotactic assays to gauge and support cellular migration in regions like the stroma (Kabak et al., 2020), and retraction methods within the endothelium and epithelium to effectively control cell adhesion and localization within the compartments (De Pieri et al., 2021). Interestingly, it is also important to highlight the use of computational modeling to guide and validate compartment-specific reseeding and cell migration.

## Evaluations of ocular function and biomarker-based assessments

### Tracking cellular differentiation using biomarkers

To observe the transitions of iPSCs into the specific corneal cell phenotype, we can track the cell-specific markers expressed by each cell type. Corneal epithelial cell markers include cytokeratin 3 (K3), cytokeratin 12 (k12), lumican, and aldehyde dehydrogenase (ALDH) (Harkin et al., 2015; Hashmani et al., 2013; Català et al., 2021). Corneal stromal cell markers include CD34, keratocans, and lumican (Li et al., 2021). Finally, corneal endothelial cell markers include N-cadherin, zona occludens-1, and Na/K-ATPase (Ju et al., 2012; He et al., 2016; Thériault et al., 2018). These markers can be identified through Western blotting or immunofluorescence. Biomarkers can be used to test the structural integrity of the graft and ensure that appropriate cells are being expressed in their locations.

### Assessing optical and structural properties of the bioengineered cornea

The eye’s cornea has the unique property of being transparent; we can measure light transmission to assess the functionality of our transplanted stem cells. At a microscopic scale, this characteristic is determined by the lack of scatter caused by corneal cells or other structures that might induce local alterations in the refractive index. On a nanoscopic level, it’s characterized by the absence of pigments or blood vessels and by the specific structure and arrangement of collagen fibrils within the lamellae (Meek and Knupp, 2015; Corridon et al., 2006). We can measure light transmission *ex vivo* and *in vivo*. The keratografts post-decellularization (Pantic et al., 2023; Wang et al., 2023b) and post-recellularization can be assessed for transparency and optical transmittance (Polisetti et al., 2021). These studies can provide insight into general ECM disruption and specific collagen fiber disorganization. Fluorescent-based microscopic evaluations and computational approaches can provide insight into cellular migration, retraction, and fiber orientation/alignment (Corridon et al., 2022; Shakeel and Corridon, 2022; Zhang et al., 2023).

## Challenges and future perspectives

After reseeding the dECM scaffold, the ideal result is a graft that mimics the natural eye and nurtures a microenvironment supporting long-term structure and function. However, a few limitations exist with using a decellularized scaffold and IPSCs for recellularization. Once the scaffold is seeded, it is hoped that intrinsic bioactive factors retained within the ECM can direct cell migration, proliferation, and differentiation. Although inducible through factors and trackable through immunofluorescence, it may be challenging to wholly control the migration and differentiation of the IPSCs. Additionally, the integrity of the ECM could be compromised via decellularization. However, the structural and functional integrity of the scaffold can be monitored and gauged through various well-known assays.

Moreover, the ECM scaffold must contain the correct tracts and pathways to facilitate the migration of native cells, such as nerves or immune cells, to reinnervate and populate our graft. A possible complication of this would be excess angiogenesis in our graft. The native cornea expresses certain factors that modulate and limit vascularization. However, cells that populate our scaffold, whether exogenous or native, risk expressing angiogenic factors that will result in blood vessel growth in the cornea, ultimately leading to reduced transparency.

## Conclusion

This approach relies on the dECM as a scaffold for xenografting, which arguably provides one of the most suitable environments for corneal regeneration. Likewise, with the bladders, the outline approach builds on previous and source pluripotent-induced stem cells from the urine to generate various cellular lineages to repopulate the three major corneal regions: the epithelium, stroma, and endothelium. The proposed pathway can potentially help develop patient-specific approaches for disease modeling, drug discovery, and further pathogenesis research while minimizing and valorizing agri-food waste.

## Data Availability

The original contributions presented in the study are included in the article/supplementary material, further inquiries can be directed to the corresponding author.
